# Does Shock Wave Application Affect the Ureteral Wall Around an Impacted Stone? A Critical Evaluation Focusing on Ureteral Wall Thickness

**DOI:** 10.3390/jcm14217636

**Published:** 2025-10-28

**Authors:** Hikmet Yasar, Salih Yildirim, Alper Asik, Emre Burak Sahinler, Gamze Simsek, Cahit Sahin, Kemal Sarica

**Affiliations:** 1 Department of Urology, Health Sciences University, Sancaktepe Training and Research Hospital, 34785 Istanbul, Turkey; yasarhikmet193@gmail.com (H.Y.); alperasik001@hotmail.com (A.A.); emre.sahinler@yahoo.com (E.B.S.); gamzemsimsek97@gmail.com (G.S.); cahitsahin129@gmail.com (C.S.); saricakemal@gmail.com (K.S.); 2 Department of Urology, Biruni University Medical School, 34015 Istanbul, Turkey

**Keywords:** ureteral wall thickness, computed tomography, extracorporeal shock wave lithotripsy, urolithiasis, proximal ureteral stones

## Abstract

**Background/Objectives**: The aim of this study was to evaluate the possible effects of shock wave (SW) application for the noninvasive treatment of impacted ureteral stones on the pericalcular ureteral tissue in terms of changes in ureteral wall thickness. **Methods**: A total of 114 patients with impacted proximal ureteral stones underwent ESWL at our department. Patient- and stone-related parameters (size, density, and location); radiological parameters, including ureteral wall thickness (UWT); and degree of hydronephrosis were assessed on NCCT images before and shortly after the procedure. The possible effects of applying high-energy shock waves to the pericalcular ureteral tissue were evaluated according to changes in ureteral wall thickness. A comparative evaluation was conducted based on the number of sessions and the outcomes of SWL. **Results**: The mean value of UWT after the first session of stone management decreased significantly when compared to the mean pre-operative value (*p* < 0.005). This was also noted after the second treatment session, after which the mean UWT was significantly lower than the pre-operative value (*p* < 0.005). However, the difference in the mean UWT before and after three sessions of SWL was not significant (*p* = 0.104). A detailed evaluation of these values in all groups revealed that although the decrease in the mean UWT in cases with a successful outcome was significant (*p* < 0.005), the change in these values was not significant in cases for which the treatment was unsuccessful (partial disintegration of the stone or no disintegration at all) (*p* = 0.145). **Conclusions**: Application of SW in patients with impacted upper ureteral stones may not have a detrimental effect on the ureteral wall or compromise a secondary procedure if the stones are successfully disintegrated and passed after one or two sessions. However, in the case of resistant stones, an increased number of sessions and more SWs may induce such adverse effects, warranting further evaluation in future studies.

## 1. Introduction

Extracorporeal shock wave lithotripsy (ESWL) is a common treatment in the management of upper urinary tract stones measuring < 10 mm in both adults and children. Due to its true noninvasive nature, high success rate, and practicality, this procedure can be performed successfully in all parts of the world if appropriate for the patient and stone condition [1,2].

Current EAU Guidelines on Urolithiasis 2024 recommend both ESWL and URS for the minimally invasive management of upper urinary tract stones. Although URS can achieve higher stone-free (SF) rates in a single session, ESWL is substantially less invasive and the treatment of choice for smaller (<10 mm) and soft (<1000 HU) proximal ureteral stones [3,4]. The reported success rates of ESWL for such stones were found to vary between 40% and 82% [5,6,7]. To further support the use of ESWL, in their detailed investigation, the EAU-AUA ureteral stone management panel indicated ESWL as the preferred management modality in the treatment of these stones [2,3].

Similar to other treatment options, the success rate of ESWL is related to certain stone characteristics, including location, size, and composition, and the stone-to-skin distance [3,4]. Additionally, if not applied in the appropriate volume and by an experienced practitioner, SWs may be associated with unexpected events.

Several studies have examined possible changes in the tissues surrounding ureteral stones due to the traumatic effects of SW [5,6,7]. These studies revealed that some histological alterations, like micro-hemorrhage, inflammation, edema formation, and fibrotic alterations, may occur in these tissues after high-energy SW application during ESWL. These alterations may be more important in the case of impacted ureteral stones, when the affected ureteral wall is exposed to the traumatic effects of SWs to a greater extent. In other words, although the aim of the treatment is to disintegrate the stone(s) located in the ureteral lumen, the ureteral wall could also be negatively affected by the SWs, and these effects may be profound if repeated treatments are required. It has been postulated that the impacted stone(s) induce irritation-related inflammatory reactions in the pericalculary ureteral wall; the possible effects of HESW in such cases require further evaluation.

The detrimental effects of SWs on the ureteral wall may affect not only the success of primary ESWL management but also the course and success of secondary ureteroscopy, if required. These effects may be more pronounced, particularly when the procedure was unsuccessful despite more than one session. Regarding this issue, ureteral wall thickness (UWT) has been thoroughly evaluated and found to be closely correlated with the severity of impaction and increased edema due to irritation induced by the stone irritation. However, despite its theoretical importance, possible alterations in the ureteral wall after SW treatment have not been well evaluated. To the best of our knowledge, our study is the first to focus on changes in the UWT after the application of SW, particularly regarding implications for the outcomes of subsequent procedures when ESWL is unsuccessful.

In this study, by evaluating changes in UWT, we aimed to assess the possible effects of applying SWs on the tissue characteristics of the pericalcular ureteral wall for patients undergoing SWL with varying numbers of sessions for stone disintegration.

## 2. Patients and Methods

This study is a retrospective observational analysis of prospectively collected clinical and radiological data obtained during routine patient management. A total of 114 adult patients (87 men and 27 women) undergoing ESWL for solitary, impacted proximal ureteral stones (<10 mm) were evaluated. Patients with active urinary tract infection, bleeding disorders, pregnancy, severe skeletal malformations, significant obesity, or previous surgical/endoscopic intervention were excluded. All patients had radiologically confirmed impacted stones without spontaneous passage despite close follow-up and appropriate medical/general measures.

UWT was measured on non-contrast CT (NCCT) images, using both axial and coronal slices. Measurements were performed with a standard soft-tissue window (width: 350 HU; level: 40 HU), applying the built-in distance tool perpendicular to the ureteral wall at the level of the stone and selecting the slice that demonstrated maximal mural thickening. Two independent radiologists (each with >5 years of experience in genitourinary imaging), blinded to the ESWL session number and treatment outcome, performed the measurements, and the mean value was used for analysis. Inter-observer reliability was excellent (ICC = 0.91, 95% CI: 0.86–0.95).

In this study, an impacted stone was defined as a ureteral calculus that remained at the same anatomic location for at least four weeks despite conservative management and medical expulsive therapy. Radiologically, impaction was confirmed by non-contrast CT (NCCT) showing a fixed calculus associated with pericalcular ureteral wall thickening (UWT ≥ 2.5 mm) and/or ipsilateral hydronephrosis.

Treatment success was defined as the absence of residual fragments > 2 mm on follow-up NCCT performed one week after the final ESWL session. Patients with residuals ≤ 2 mm were categorized as having clinically insignificant residual fragments (CIRFs) and considered stone-free according to EAU criteria.

Baseline NCCT and follow-up scans were evaluated independently by two radiologists blinded to the number of sessions and treatment outcome.

All patients underwent non-contrast CT (NCCT) imaging within 48 h before the first ESWL session (baseline) and one week after the final session (follow-up). Both scans were obtained on the same 128-slice multidetector CT scanner (Siemens SOMATOM Definition AS, Erlangen, Germany), using identical acquisition parameters (120 kVp; 200–250 mAs; 1.25 mm slice thickness; soft-tissue kernel reconstruction). All images were evaluated using a standard soft-tissue window (width 350 HU, level 40 HU). Both scans were acquired under low-dose protocol settings in accordance with the ALARA principle.

To minimize variability, identical patient positioning and scan range were maintained. However, we recognize that temporal factors—such as the natural resolution of ureteral wall edema over time—may have influenced post-treatment measurements, which we have noted as a limitation.

The patients were divided into three groups based on the number of ESWL sessions required for a successful outcome: Group 1 (*n* = 42) required one session, Group 2 (*n* = 40) required two sessions, and Group 3 (*n* = 32) required three sessions. Another group included patients for whom treatment was unsuccessful despite three sessions. Treatment success was defined as complete stone clearance on follow-up radiological imaging.

Non-contrast computed tomography (NCCT) was performed in the beginning and one week after the final ESWL session. Ureteral wall thickness (UWT) was measured at the level of the stone in axial and coronal NCCT sections. Changes in UWT on pre- and post-treatment imaging were compared within each group.

ESWL was performed using a Modulith SLK inline lithotripter (Storz Medical, Switzerland), with a maximum of 3000 shocks per session at 14–22 kV and a frequency of 60–90 shocks per minute. All procedures were performed under NSAID analgesia. Changes in the upper tract were followed-up with using KUB and ultrasound evaluation, while NCCT was performed to evaluate changes in the pericalcular ureteral wall (in terms of UWT) one week after the last ESWL session.

Statistical analysis was conducted with a paired samples t-test to evaluate the significance of pre- and post-treatment UWT differences. *p* values < 0.05 were considered statistically significant.

Ethical approval for this study was obtained from the Sancaktepe Training and Research Hospital Ethics Committee on 30 April 2025 (Approval No: 133).

## 3. Results

A total of 114 patients with a mean age of 45 years (23–74 years) were included. All had impacted proximal ureteral stones < 10 mm, with a mean stone size of 8.92 ± 2.45 mm. The mean BMI value was 26.65 ± 4.71, and no patient had extreme obesity, which can affect the effects of SWs on the stone and ureteral wall.

Patient demographic characteristics and the results of a comparative evaluation of stone-related factors before and after the ESWL sessions are provided in Table 1.

Our results revealed the following findings:

Mean UWT values, which are supposed to reflect the presence and severity of edema in the ureteral wall, decreased in a statistically significant manner for cases in which the patients were completely SF after one or two sessions (*p*: < 0.005 and *p*: < 0.005, respectively). However, the mean UWT did not change and maintained its high values in patients who required three sessions (*p*: 0.104) for successful stone disintegration. The same was true for patients in whom SWL was unsuccessful despite three sessions (*p*: 0.145). These findings call into question the limited (potentially positive) traumatic effect of high-energy SWs on the ureteral wall in patients who are SF after one or two sessions and receiving fewer SWs.

In multivariable ANCOVA, the post-ESWL UWT was independently associated with session number and baseline UWT. Patients who were stone-free after one or two sessions showed significantly lower adjusted post-treatment UWT values compared with those requiring three sessions (*p* < 0.05). Larger stones and a higher baseline UWT were correlated with thicker post-treatment ureteral walls. In multivariable logistic regression, session number and stone size were the strongest predictors of stone-free status. Patients who achieved clearance following the first or second session had markedly higher adjusted odds of success, whereas a higher HU and larger stones were negatively associated with stone-free outcomes (Table 2).

## 4. Discussion

Management of proximal ureteral calculi, in which ESWL and ureteroscopy (URS) are commonly applied modalities, remains challenging for practicing urologists [8]. The EAU and AUA guidelines recommend both procedures equally [9]. As a noninvasive procedure, ESWL has been found to be effective for the management of calculi measuring < 10 mm [10,11]. Although successful outcomes are often achieved after a single session (with the use of smaller scopes and Ho-YAG laser technology), URS is more invasive than SWL and may be associated with severe complications, especially when performed by inexperienced practitioners [5].

Regarding the effective application of ESWL for impacted proximal ureteral calculi, some stone- (size, location, and density) and patient-related (body habitus, anatomical characteristics of the upper urinary tract, and corporeal deformities) factors can influence the final success rate [3,4,5]. Therefore, the decision to use ESWL must be based on the above-mentioned factors, as adequate stone disintegration may not be accomplished, as indicated by limited SF rates even after several sessions. As a result, patients with ESWL-resistant stones may require a secondary procedure, usually URS, to achieve SF status.

A comparative evaluation of the outcomes obtained using both approaches revealed that ESWL success rates for proximal ureteral stones vary between 40% and 82%, and the URS success rate is higher [5,6,7]. As mentioned above, the majority of the patients with smaller and softer stones (<10 mm and <1000 HU) are good candidates for SWL [6]. Despite ESWL’s noninvasive nature and success in such stones, the risk of SW-induced complications, along with possible changes in the tissues surrounding the stones, require serious consideration. Studies focusing on this issue have demonstrated that cavitation induced by SW application, hydrodynamic jet flows, and the formation of free oxygen radicals in the tissue exposed to this type of trauma may induce certain effects in the tissue cells by changing their cell membrane permeability [12,13]. Additionally, SWs were found to induce acute ureteral mucosal damage during the disintegration of ureteral stones [14,15]. Thus, a thorough radiological evaluation to evaluate such possible traumatic effects of SWs on the ureteral wall surrounding the impacted calculus is needed, particularly for patients undergoing several SWL sessions [6,11,12].

It is clear that during the management of impacted proximal ureteral stones, the application of high-energy SWs (particularly in when repeated) may induce certain changes in the wall (and mucosa) of the ureter surrounding the embedded stone. This issue gains importance when considering that stones located in the ureter may induce inflammation and irritation, which may result in edema and polypoid formations on the ureteral wall. Taking these facts into account, the possible additional effects of SWs on the ureteral wall require further clarification. Studies on the effects of SWs on the ureteral wall and their possible impact on the outcome of auxiliary URS applications in patients unresponsive to SWL are extremely limited [16,17].

The outcomes of studies addressing this issue have revealed that some histological events, like micro-hemorrhage, inflammation, edema formation, and fibrotic alterations, may occur in tissues exposed to SWs during SWL [18,19]. These alterations may be of further importance for patients with impacted ureteral stones, where the thin ureteral wall is exposed to the traumatic effects of SWs to a greater extent, particularly in patients undergoing repeated sessions. Additionally, the course and success of secondary procedures, particularly ureteroscopy, could be affected by these alterations [20].

Regarding this issue, UWT has been assessed to evaluate the degree of stone impaction, as it is a highly correlated parameter. However, despite its evident importance, possible alterations in the ureteral wall following exposure to SWs have not been well evaluated thus far. Our study is the first to focus on changes in the UWT after SWL, which could affect the course of subsequent procedures if SWL is insufficient for stone disintegration.

In their original study, Kılınc MF et al. showed that previous use of SWL might not affect the outcome of URS and has very low major complication rates, similar to those of primary URS [20]. In another study, Akkas F et al. identified UWT as a predictor of stone impaction and its role in choosing the appropriate treatment modality. Although that study also mentioned the possible effect of stone impaction on the performance of URS after ESWL, it did not directly evaluate the effects of SW application on the ureteral wall [21].

Regarding changes in tissue edema in cases where ESWL was successful, eliminating the stone from the ureter seems to be main driver for the resolution of the edema, which was induced by the stone embedded into the ureteral wall. However, we believe that ESWL exposure may still contribute to tissue effects in non-responders by limiting the degree of edema formation in these cases.

Successful stone clearance following ESWL was associated with a decrease in pericalcular ureteral wall thickness, likely reflecting the resolution of obstruction-related edema rather than a direct effect of the shock waves. Assessment of UWT before and after the treatments clearly demonstrated a significant decrease in UWT in patients who were SF after one and two sessions. In other words, in these cases, SWL acted by decreasing the extent of edema to allow the stones to be disintegrated and passed after treatment. The mean UWT value did not change significantly in unsuccessful cases, where SF status was not attained despite three treatment sessions. This indicates the persistence of ureteral wall edema, which prevented the SWs from affecting stone disintegration and spontaneous passage.

These findings raise questions about the limited (potentially positive) traumatic effects of HESW on the ureteral wall in patients who require fewer SWL sessions to become SF. Of course, eliminating the stones from the ureteral lumen (the main cause of irritation and edema formation) may also have a certain effect on the decrease in ureteral wall edema. This finding could also indicate a lack of negative effects of applying SWs in these cases, which some authors have proposed are a critical factor that may worsen the application and outcomes of subsequent ureteroscopy.

The multivariable analysis demonstrated that successful and early stone disintegration is the key determinant of decreased ureteral wall thickness following ESWL. After adjusting for baseline UWT, stone size, density, and patient-related variables, only session number remained independently associated with post-treatment wall thickness. This finding suggests that the resolution of obstruction and inflammation, rather than the direct effect of shock waves, drives the reduction in pericalcular edema.

Similarly, the logistic regression model confirmed that patients who achieved stone clearance after one or two sessions had substantially higher odds of being stone-free, while greater stone size and higher HU values were inversely related to success. These observations are consistent with previous studies indicating that stone burden and density significantly influence ESWL efficacy.

Importantly, the consistent association between early clearance and a thinner ureteral wall supports the notion that timely stone removal mitigates chronic ureteral wall changes. Our results highlight that persistent obstruction after multiple ESWL sessions may maintain or even exacerbate local wall thickening, potentially explaining lower success rates in these patients.

Based on our current findings indicating a decrease in UWT, we conclude that the unnecessary application of SWs in patients with an unsuccessful outcome despite two sessions may be associated with changes in ureteral wall morphology. As a result, the outcomes of both SWL and secondary procedures like ureteroscopy could be compromised. Thus, SWs may not induce undesired changes in the ureteral wall if indication to perform ESWL is appropriate and the stones are disintegrated following one or two sessions. Last but not least, in these cases, ureteroscopy after SWL may not be complicated, since no evident change in the ureteral wall was noted in our cases.

This study has several limitations. First, it was conducted at a single center, which may limit the generalizability of the results. Second, although the overall sample size was acceptable, subgroup analyses (e.g., patients requiring three sessions or those with unsuccessful outcomes) included relatively few patients, which may affect statistical power. Third, the follow-up period was limited to three months, potentially missing long-term effects or late complications. Additionally, UWT was evaluated using NCCT only, without histological confirmation or alternative imaging methods to differentiate between edema and other causes of thickening, such as fibrosis. Finally, despite standardized treatment protocols, individual patient factors—such as anatomy, stone composition, and body mass index—could have influenced both treatment outcomes and the measurement of UWT values. Although all CT scans were performed with identical technical parameters, the interval between sessions may have allowed for partial spontaneous regression of pericalcular edema, representing a potential temporal bias. However, taking into account the very limited data available in the literature on this critical issue, we believe that our current findings contribute sufficiently to existing scholarship. Further multicenter studies in larger populations and with longer follow-up periods are needed to confirm our findings.

## 5. Conclusions

The application of SWs in patients with impacted upper ureteral stones may not have a detrimental effect on the ureteral wall or compromise the outcome of secondary procedures if the stones are successfully disintegrated and passed after one or two sessions of SWL. For patients with SW-resistant stones, a higher number of sessions may induce adverse effects and compromise the course of SWL, as well as secondary procedures like ureteroscopic lithotripsy. Early and successful stone clearance is independently associated with decreased ureteral wall thickness, suggesting that the resolution of obstruction rather than shock wave exposure itself drives the improvement in ureteral wall morphology. However, we believe that further studies, particularly in cases requiring repeated sessions, are needed.

## Figures and Tables

**Table 1:** Comparison of demographic, clinical, and radiological parameters across patient groups stratified by number of ESWL sessions.

Variable	1 Session (42/114)	2 Session (40/114)	3 Session (32/114)	*p* Value
**Age** (Years)	39.31 ± 9.96	45.10 ± 14.48	43.88 ± 10.86	0.076
**Gender**				
Male	32 (76.2%)	28 (70%)	27 (84.4%)	
Female	10 (23.8%)	12 (30%)	5 (15.6%)	0.362
**BMI**	26.82 ± 3.49	26.94 ± 4.35	27.19 ± 5.9	0.942
**Comorbidities**				
Non	29 (69.04%)	16 (40%)	20 (62.5%)	
HT	5 (11.9%)	6 (15%)	5 (15.6%)	0.88
DM	4 (9.5%)	4 (10%)	5 (15.6%)	0.674
Other	4 (9.5%)	4 (10%)	2 (6.3%)	0.514
**Family History**				
Yes	25 (59.5%)	22 (60%)	19 (68.8%)	
No	17 (40.5%)	18 (40%)	13 (31.3%)	0.801
**Ureteral Location**				
Upper	28 (66.7%)	22 (55%)	13 (40.6%)	
Middle	6 (14.3%)	10 (25%)	6 (18.8%)	
Lower	8 (19%)	8 (20%)	13 (40.6%)	0.112
**Radiologic Parameters**				
**Hounsfield Unit**	754.12 ± 99.21	904.32 ± 42.39	928 ± 67.08	0.006
**Stone-Skin Distance**	116.2 ± 17.48	117.22 ± 16.45	116.99 ± 12.8	0.960
**Stone Size (mm)**	8.55 ± 2.3	9.29 ± 2.3	9.28 ± 2.39	0.277
**Stone Side**				
Left	22 (52.4%)	18 (45%)	17 (53.1%)	
Right	20 (47.6%)	22 (55%)	15 (46.9%)	0.733
**Ureteral Wall Thickness**				
Before Treatment	3.33 ± 1.04	3.86 ± 1.13	3.29 ± 1.12	
After Treatment (1 week)	2.54 ± 1.19	2.69 ± 0.87	2.95 ± 1.19	
Change	0.79 ± 1.07	1.16 ± 1.49	0.34 ± 1.15	0.024
**Hydronephrosis Grade**				
Grade 0	11 (26.2%)	6 (15%)	2 (6.3%)	
Grade 1	20 (47.6%)	14 (35%)	11 (34.4%)	
Grade 2	10 (23.8%)	12 (30%)	17 (53.1%)	
Grade 3	1 (2.4%)	20 (20%)	2 (6.3%)	0.007
**Treatment Success**				
Successful	38 (90.5%)	30 (75%)	26 (81.3%)	
Residual Stone	1 (2.4%)	6 (15%)	2 (6.3%)	
Failure	3 (7.1%)	4 (10%)	4 (12.5%)	0.245
**Ureteral Catheter**				
Yes	6 (14.3%)	10 (25%)	3 (9.4%)	
No	36 (85.7%)	30 (75%)	29 (90.6%)	0.183

**Table 2:** Multivariable ANCOVA assessing independent predictors of post-ESWL ureteral wall thickness (UWT) after adjustment for baseline UWT and stone-related parameters.

Predictor	aOR	95% CI (Low)	95% CI (High)	*p*-Value
2 sessions vs. 1 session	1.42	0.24	8.42	0.696
3 sessions vs. 1 session	0.62	0.10	3.85	0.610
Female vs. male	0.73	0.15	3.65	0.701
Pre UWT	1.04	0.50	2.16	0.908
Stone size	0.79	0.59	1.06	0.114
HU	1.00	1.00	1.00	0.083
SSD (stone skin distance)	1.03	0.97	1.09	0.291
Hydroneprosis	0.94	0.38	2.34	0.900
BMI	0.85	0.67	1.08	0.177
Age	0.94	0.87	1.01	0.070

aOR (adjusted odds ratio), CI (confidence interval).

## Data Availability

The original contributions presented in this study are included in the article. Further inquiries can be directed to the corresponding author(s).
